# Utilization and Staff Perspectives on an On-Demand Telemedicine Model for People with Intellectual and Developmental Disabilities Who Reside in Certified Group Residences

**DOI:** 10.1089/tmr.2023.0024

**Published:** 2023-07-31

**Authors:** Carolyn A. Berry, Lorraine Kwok, Miriam Gofine, Matthew Kaufman, Debra A. Williams, Kelly Terlizzi, Mike Alvaro, Charles J. Neighbors

**Affiliations:** ^1^ Department of Population Health, New York University Grossman School of Medicine, New York, New York, USA.; ^2^ StationMD, Maplewood, New Jersey, USA.; ^3^ Cerebral Palsy Associations of New York State, Cohoes, New York, USA.; ^4^ Department of Surgery, New York University Grossman School of Medicine, New York, New York, USA.

**Keywords:** developmental disability, emergency department, intellectual disability, telemedicine, qualitative research

## Abstract

**Background:**

Non-emergent medical problems that arise when a usual provider is unavailable can often result in emergency department or urgent care visits, which can be particularly distressing to people with intellectual and developmental disabilities (PIDD). On-demand, synchronous telemedicine may be a promising supplement when immediate care from usual sources is unavailable. Prior research demonstrated that high-quality telemedicine can be effectively delivered to PIDD. The aim of this article is to describe the utilization and staff perspectives on the implementation of the Telemedicine Triage Project (TTP), an innovative model that provides telemedicine consultations for PIDD who reside in state-certified group residences and present with an urgent but non-emergent medical concern when their usual provider is unavailable.

**Methods:**

Call frequency data for calendar years 2020 and 2021 were reviewed. The study team conducted semi-structured interviews, with 19 key informants representing organizational- and agency-level leadership and staff. The interview data were analyzed using a protocol-driven, rapid qualitative methodology.

**Results:**

Telemedicine consultations increased from 7953 in 2020 to 15,011 calls in 2021, and call volume peaked between 10 am and 1 pm. Key informants reported high satisfaction with TTP; universal benefits and a few barriers to implementation; and strong interest in maintaining the program beyond the grant period.

**Discussion:**

Over the first 2 years of its implementation, the TTP program was widely utilized and proved extremely feasible and acceptable to staff. This model is a promising and highly feasible way to provide equitable access to telemedicine for PIDD by addressing barriers to and disparities in access to health care that affect PIDD.

## Introduction

Accessing health care services can be challenging for people with intellectual and developmental disabilities (PIDD) due to high costs for medical care and prescription medication; lack of transportation to and from health care facilities; physical inaccessibility of facilities; and difficulty communicating with health care providers.^1,2^

Moreover, in a recent qualitative study, community-based primary care physicians (PCPs) and specialists identified barriers to providing care for people with disabilities, including lack of knowledge, experience, and skills to provide such care; inaccessible clinics and equipment; and challenges with accommodating communication needs (e.g., cost of providing sign language interpretation services).^3^

Several physicians also noted that people with disabilities made up a small portion of their overall caseload, implying that there was little need to provide physical or communication accommodations.^3^

Although emergency department (ED) visits are stressful to anyone, they can be particularly distressing to PIDD because of long ED wait times,^4,5^ inaccessible medical facilities, discriminatory or insufficiently culturally competent providers,^5–9^ and unavailability of individualized accommodations.^6,10^ These factors can worsen the overall experience of the ED visit for PIDD and lead to unnecessary and costly assessments and treatments.^5,11^

Visits to EDs are also logistically complicated and financially costly to agencies managing group residences, which are required to maintain state-mandated staffing ratios within the residences.^12^ With a staff member accompanying a resident to the ED, the agency must arrange for staffing at the residence. Thus, minimizing unnecessary ED visits for PIDD in group residences should be a high priority.

A non-emergent medical problem that arises when a PCP or specialist who usually provides care is unavailable, for example after office hours, can create a very challenging situation.^13–15^ Although the drivers of observed patterns of ED use/overuse by PIDD are under-researched,^4,16^ anecdotal evidence suggests that these situations often result in ED visits.

Lack of access to urgent care (UC)^†^ may be among the drivers of the finding that PIDD use the ED at significantly higher rates than the general population,^4,17^ especially to address concerns that could be treated in outpatient or primary care settings.^6,16,18,19^ The PIDD who reside in group residences are more likely than the general population to require after hours guidance from a PCP.^15^ However, there is minimal research on after-hours health care for PIDD that occurs outside of the ED,^13,14,20^ and, to our knowledge, no such research conducted in the United States.

On-demand, synchronous telemedicine may be a promising supplement to usual care when non-emergency medical problems arise after-hours or when immediate care is otherwise unavailable.^21,22^ Telemedicine, a subset of telehealth, refers to the use of electronic information and communication technologies to provide health care at a distance.^23,24^ Although synchronous telemedicine increased dramatically during the COVID-19 pandemic,^25,26^ it is critical to identify telemedicine initiatives that maximize its known benefits to underserved populations, including PIDD.^27^

Prior research indicates that high-quality, accessible telemedicine can be effectively delivered to PIDD.^27^ When deployed in the appropriate context, telemedicine delivered to this population is associated with positive outcomes, including reduced travel time and costs for patients and caregivers;^27,28^ reduced health care costs; and improved health outcomes, including reduced hospitalizations and ED visits.^27^ Further, accessing care in a familiar environment may also make the appointment less stressful to PIDD and facilitate a productive clinical encounter.^27–30^

The aim of this article is to describe the utilization and staff perspectives on the implementation of the Telemedicine Triage Project (TTP), an innovative model that provides telemedicine consultations for PIDD who reside in state-certified group residences across the state of New York in the United States, and present with an urgent but non-emergent medical concern when their usual provider is unavailable.

### Telemedicine triage project

The TTP is funded by a 5-year New York State Department of Health Statewide Health Care Facility Transformation Program grant to Cerebral Palsy Associations of New York State (CPNYS) from 2019 to 2024. CPNYS is a multi-service non-profit organization that provides resources to >100,000 PIDD and their families.^31^ CPNYS manages TTP and contracts with StationMD, a for-profit physician service that provides telemedicine to PIDD and other vulnerable populations.^32^

Despite its name, TTP is not only a triaging service^‡^; TTP also enables 24/7 access to telemedicine provided by board-certified providers (primarily emergency medicine) who are specially trained to address PIDD's complex health needs. One component of the encounters helps to determine the necessary level of care (e.g., if a patient requires transport to an ED) and another is the delivery of medical services themselves, when this can be appropriately done via telemedicine.

Nurses who typically cover several residences are available during weekday work hours, and on-call nursing is available after hours and on weekends. Residents have primary care and specialty providers based in federally qualified health centers affiliated with some of the agencies, or in other community-based clinics; no primary or specialty care is available on-site at the residences. The program is explicitly intended to complement—not replace—residents' usual care by providing access to alternative physicians when usual providers are not available. The implementation of TTP in group homes began in the last months of 2019 and ramped up statewide in in the early months of 2020.

The TTP is offered in 1153 state-certified group residences across NYS, which collectively house >8000 residents. All participating group residences are affiliated with one of CPNYS' 18 agency affiliates, The Arc New York's 24 agency affiliates, or 7 other agencies that CPNYS invited to participate. Participating residences serve adults and children with intellectual and developmental disabilities.

The complexity of residents' health conditions varies from residence to residence; some residents are older, have difficulty with their mobility, and have a high number of medical diagnoses that need to be managed whereas others are younger and have fewer medical needs. Consent for participation in TTP's telemedicine services is provided by residents' family members/guardians; the refusal of consent is quite rare.

StationMD installed kiosks and/or tablet-based workstations equipped with Bluetooth-enabled devices such as stethoscopes in residences. Agency staff participate in initial and on-going training (as needed), including practice sessions with StationMD trainers. A 4-min training video, a step-by-step written guide, and phone-based technical support are consistently available.

The TTP fits into existent agency on-call nursing workflows, working in conjunction with agency/residence nurses, who are either agency staff or a contracted vendor. In the case of a life-threatening emergency, direct support professionals (DSPs) at the residences call 911 immediately. As per NYS regulations, DSPs are required to contact the on-call nurse before initiating a telemedicine visit with StationMD.

The nurse assesses the situation and directs the DSP to either independently address the problem; arrange an ED visit; or initiate a telemedicine consultation with StationMD. Agency nurses and residents' family members/guardians may also join the consultations. Telemedicine consultations occur through an HIPAA-compliant StationMD-developed platform available on a mobile app, a tablet, or laptop.

The DSP provides vital signs to the StationMD physician and assists the resident as needed during the video call. The StationMD physician may decide to send the resident to the ED or resolve the medical situation with instructions to the DSP, a new prescription, or medication refill.

## Methods

All study protocols and procedures were approved by the New York University Grossman School of Medicine Institutional Review Board.

### Quantitative data

CPNYS and StationMD provided the call frequency data for calendar years 2020 and 2021, including monthly data on chief complaint, International Classification of Diseases (ICD)-10 diagnoses codes and descriptions, and the number of calls: per agency; by day of week; by hour of day; by outcome (categorized by physician recommendation to transfer to ED/UC or not); and per capita per agency.

### Qualitative data

#### Data collection

Two members of the research team conducted semi-structured interviews (see Supplemental File for interview protocols) with a purposive sample of 19 key informants representing organizational- (CPNYS and StationMD) and agency-level (participating CPNYS affiliate agencies, The Arc of New York affiliate agencies, and independent agencies) leadership and staff (DSPs and nurses), between February and June 2021.

The research team did not conduct interviews with residents, because that was outside the scope of work for this preliminary assessment of the implementation of TTP. Participation in the interviews was voluntary, and participants provided informed consent verbally. Interviews lasted 20–60 min and explored program history, current operations, and sustainability; facilitators and barriers to the implementation of TTP; and suggestions for improvement.

Interviews were audio-recorded with participants' permission and transcribed. Interviewees did not receive incentives for participation. The research team reviewed relevant program documents provided by CPNYS and StationMD.

#### Data analyses

Interview data were analyzed using a protocol-driven, rapid qualitative methodology.^33^ The research team developed a summary template and assigned each interview question a pre-determined domain name in the template. Domains included facilitators, barriers/challenges, benefits, and the sustainability of TTP implementation; suggestions to improve TTP; and COVID-19 related effects on TTP. The team reviewed the summary template and then divided the completion of a summary for each interview transcript within the group. Summaries captured each domain's key elements and illustrative quotes. The team synthesized data within domains and identified emergent themes across interviews using a findings matrix.

## Results

### Utilization of TTP in 2020 and 2021

Table 1 shows the number of group residences, residents, calls per agency, and calls per resident by agency in 2020, the first full year of implementation, and 2021. The number of residences and residents per agency remained stable between 2020 and 2021. Telemedicine consultations increased from 7953 in 2020 to 15,011 calls in 2021.

**Table 1.** Number of Certified Group Residences, Residents, and Calls per Agency in 2020 and 2021

Agency	Number of certified group residences	Number of residents in 2020 and 2021	Number of calls 2020	Number of calls per resident 2020	Number of calls 2021	Number of calls per resident 2021
Agency 1	10	74	270	3.65	266	3.59
Agency 2	25	113	19	0.17	26	0.23
Agency 3	14	219	66	0.30	96	0.44
Agency 4	78	602	230	0.38	283	0.47
Agency 5	8	67	172	2.57	214	3.19
Agency 6	43	323	22	0.07	29	0.09
Agency 7	5	31	17	0.55	35	1.13
Agency 8	38	301	0	0.00	140	0.47
Agency 9	7	66	125	1.89	107	1.62
Agency 10	10	71	147	2.07	135	1.90
Agency 11	21	120	60	0.50	263	2.19
Agency 12	8	93	67	0.72	170	1.83
Agency 13	6	49	19	0.39	40	0.82
Agency 14	6	64	86	1.34	110	1.72
Agency 15	25	181	99	0.55	190	1.05
Agency 16	10	59	26	0.44	47	0.80
Agency 17	103	587	1036	1.76	1174	2.00
Agency 18	19	102	114	1.12	389	3.81
Agency 19	78	548	225	0.41	734	1.34
Agency 20	45	338	505	1.49	780	2.31
Agency 21	35	280	674	2.41	941	3.36
Agency 22	10	101	48	0.48	48	0.48
Agency 23	30	208	42	0.20	190	0.91
Agency 24	47	406	1497	3.69	3932	9.68
Agency 25	14	90	21	0.23	31	0.34
Agency 26	16	100	129	1.29	212	2.12
Agency 27	30	207	69	0.33	46	0.22
Agency 28	41	213	24	0.11	67	0.31
Agency 29	9	124	6	0.05	0	0.00
Agency 30	9	36	27	0.75	59	1.64
Agency 31	33	153	99	0.65	178	1.16
Agency 32	10	59	104	1.76	48	0.81
Agency 33	16	107	54	0.50	79	0.74
Agency 34	10	55	22	0.40	41	0.75
Agency 35	9	78	38	0.49	35	0.45
Agency 36	17	181	65	0.36	93	0.51
Agency 37	7	59	20	0.34	6	0.10
Agency 38	12	77	31	0.40	95	1.23
Agency 39	21	128	156	1.22	169	1.32
Agency 40	22	127	137	1.08	218	1.72
Agency 41	51	361	777	2.15	1715	4.75
Agency 42	42	307	216	0.70	398	1.30
Agency 43	14	106	75	0.71	120	1.13
Agency 44	18	131	159	1.21	126	0.96
Agency 45	7	53	6	0.11	0	0.00
Agency 46	3	12	0	0.00	2	0.17
Agency 47	18	127	122	0.96	544	4.28
Agency 48	28	198	30	0.15	378	1.91
Agency 49	15	84	0	0.00	12	0.14
**Total**	**1153**	**8176**	**7953**	**0.97**	**15,011**	**1.84**

Residence staff reported chief complaints to StationMD before the telemedicine consultation during both time periods and most commonly included: medication refills or changes; abdominal pain; abrasions to various parts of the body; behavioral issues (e.g., self-injury, biting); fever; or reviewing X-ray results. Table 2 presents the most common ICD-10 diagnoses coded by StationMD physicians in 2020 and 2021 for residents with a telemedicine consultation.

**Table 2.** Most Common International Classification of Diseases-10 Diagnoses (Code and Description) in 2020 and 2021

Year	ICD-10 code	Description	Number of diagnoses (%)
2020	Z760/Z76.0	Encounter for issue of repeat prescription/medication refills	729 (9.2)
	Z7689/Z00.00/Z0000^a^	Persons encountering health services in other specific circumstances/encounter for general adult medical examination without abnormal findings	825 (10.4)
	R21	Rash and other nonspecific skin eruption	362 (4.6)
	R50.84	Febrile nonhemolytic transfusion reaction	245 (3.1)
	J06.9	Acute upper respiratory infection, unspecified	200 (2.5)
	R05	Cough	182 (2.3)
	L98.9	Disorder of the skin and subcutaneous tissue, unspecified	171 (2.2)
	L03.90	Cellulitis, unspecified	139 (1.7)
2021	Z7689/Z0000^a^	Persons encountering health services in other specific circumstances/encounter for general adult medical examination without abnormal findings	2120 (14.1)
	Z760	Encounter for issue of repeat prescription	1590 (10.6)
	Z20822	Contact with and (suspected) exposure to COVID-19	493 (3.3)
	L98.9	Disorder of the skin and subcutaneous tissue, unspecified	485 (3.2)
	Z20828	Contact with and (suspected) exposure to other viral communicable diseases	420 (2.8)
	R21	Rash and other nonspecific skin eruption	403 (2.7)

^a^ Residents served by StationMD often present with medical needs that are specific to people with intellectual and developmental disabilities, and are not encountered by the general population. These include medication problems (e.g., inadvertent overdosing, administering the wrong medication), mandated medical clearance (e.g., falls, altercations without injury), and staff questions about how to use adaptive devices or medical equipment such as wheelchairs. To document the telemedicine consultation and visit reason, StationMD physicians assign a general ICD-10 code (i.e., Z7689, Z00.00, Z0000).

ICD, International Classification of Diseases.

The most common diagnoses in 2020 included: repeat prescription; medication refills; health services in other specific circumstances; adult medical examination without abnormal findings; and rash. The most common diagnoses in 2021 included: health services in other specific circumstances; adult medical examination without abnormal findings; repeat prescription; and contact with and suspected exposure to COVID-19.

Eleven percent of calls (*n* = 837) in 2020 and 6% (*n* = 895) in 2021 resulted in transfer to the ED or UC. Call volume in both years peaked between 10 am and 1 pm. Table 3 presents the most common ICD-10 diagnoses for calls that resulted in transfers in 2020 and 2021.

**Table 3.** Most Common International Classification of Diseases-10 Diagnoses (Code and Description) Among Cases Transferred to the Emergency Department/Urgent Care in 2020 (*n* = 837) and 2021 (*n* = 895)

Year	ICD-10 Code	Description	Number of diagnoses (%)
2020	R10.9/R109	Unspecified abdominal pain	72 (8.6)
	R41.82/R4182	Altered mental status, unspecified	55 (6.6)
	S09.90XA	Unspecified injury of head, initial encounter	28 (3.3)
	R09.02	Hypoxemia	25 (3.0)
	R07.9	Chest pain, unspecified	22 (2.6)
	A41.9	Sepsis, unspecified organism	20 (2.4)
	R50.84	Febrile nonhemolytic transfusion reaction	20 (2.4)
2021	R109	Unspecified abdominal pain	48 (5.4)
	S09.90XA	Unspecified injury of head, initial encounter	46 (5.1)
	R09.02	Hypoxemia	40 (4.5)
	R1110	Vomiting, unspecified	37 (4.1)
	R07.9	Chest pain, unspecified	33 (3.7)
	R41.82	Altered mental status, unspecified	32 (3.6)
	M79.609	Pain in unspecified limb	27 (3.0)
	R509	Fever, unspecified	23 (2.6)
	G40.89	Other seizures	20 (2.2)
	I9589	Other hypotension	20 (2.2)
	R0603	Acute respiratory distress	20 (2.2)

### Goals of TTP

Despite the name of the initiative, TTP is not merely a triaging service; StationMD physicians provide direct telemedicine services when usual providers are unavailable. Further, one member of organizational-level leadership, one member of agency-level leadership, and one residential staff member observed that although TTP was not designed to provide routine care services to residents, staff used StationMD to “fill in the gaps” for some situations that normally would have required an office visit, such as renewing prescriptions, or when a resident was not allowed to enter a clinic because the resident could not tolerate a mandatory face mask.

*This is not a replacement for primary care, but frankly, we're finding that as the COVID pandemic has made its way through it has been used for some stopgap measures that probably should have gone to primary care. Because of the clinicians and the need for whether it's a script or something else that they would've gone to the ER for because they couldn't get to their doctor, it's become more than just a triaging. It's sort of a borderline wraparound to the primary care for some of these agencies.* (Executive Director)

*I think the biggest benefit is… we have someone that we can get ahold of if we don't have a primary care provider available, which in turn provides safety, really, to our individuals. They have a doctor available 24/7 for recommendations or anything like that.* (Residential RN)

### Facilitators to implementing TTP

Interviewees almost universally agreed that StationMD's trainings; administrative flexibility and responsiveness; and physicians' good bedside manner and effective communication were key facilitators of TTP's implementation. One member of organizational-level leadership, four members of agency-level leadership, and three residential staff members acknowledged the ease of using StationMD's kiosks/workstations and devices and the high quality of StationMD's training materials. One residential team leader noted that StationMD's ongoing refresher trainings adequately accommodated residences with high staff turnover.

*There was a training online that all the staff had to do. And there's also a book that we have in with the Station MD computer that is step-by-step walkthrough if, for some reason, we had a staff that didn't go through the training yet. But it's pretty easy to use.* (Residential Team Leader)

Two members of organizational-level leadership and three members of agency-level leadership noted that StationMD staff were very flexible and responsive when addressing not only their residents' needs but also their staffs' requests and questions, which was particularly valuable during the height of the COVID-19 pandemic.

*They've been very forthcoming, as far as when we asked about coming out to do additional training, they were willing to do that … Anything I need, I feel they're very responsive.* (Vice President)

One organizational-level leader and two agency-level leaders also noted that StationMD's physicians exhibited good bedside manner and “personal touch” when seeing residents.

The physicians communicated effectively with DSPs, who often do not have formal clinical training.

*They've been so good with our staff. They understand that they're not medical professionals, so they communicate really well with them. And sometimes I feel like if they went to an urgent care or the hospital they expect them to know.* (Residential RN)

According to leadership and staff, the sudden emergence of the COVID-19 pandemic necessitated the quick adoption of TTP in most residences. Staff who had been hesitant and/or skeptical about TTP before the pandemic had no choice but to use the service. Many became more comfortable with the technology over time and repeated positive experiences.

### Barriers/challenges to implementing TTP

Leadership and staff identified few barriers to implementing TTP in residences. Challenges with the equipment and technology included unreliable Internet service and keeping the equipment fully charged and ready for use.

One member of organizational-level leadership, one member of agency-level leadership, and one residential staff member reported the staff's initial hesitancy to adopt the telemedicine technology, which was new to many of them. Three interviewees from organizational leadership specifically indicated that high staff turnover at the residences necessitated continuous onboarding for when and how to use the StationMD platform.

Some DSPs were also reluctant to call StationMD as a result of prior negative experiences when working with physicians.

*I think at first they were hesitant. And, again, that kind of – in my opinion – falls back to maybe bad experiences with doctors in the past… You get treated differently. You're expected to know all of these things, and you don't. Sometimes you have to be like, hold on a minute. They're not a medical professional. They're not even – they have no medical education besides the papers we give them to read.* (Residential RN)

Because it was rare for family members/guardians not to provide consent for a resident's participation in TTP, one member of agency-level leadership noted that it was an added challenge to keep track of which residents' family members/guardians did not consent to receiving services from StationMD.

One member of organizational leadership found the state requirement for DSPs to contact the agency nurse before calling StationMD to be unnecessary and burdensome to nurses.

One DSP noted that they have not needed to call StationMD, because their residents were healthy and one agency-level leader noted that the residents' usual providers offered telemedicine services during the day and were available by phone after-hours. Moreover, one member of organizational-level leadership mentioned that some residences were too overwhelmed by COVID-19 to integrate TTP into their workflows.

One agency-level leader specifically mentioned that the pandemic forced their agency's federally qualified health center to rapidly adopt telemedicine, which, in turn, allowed residents to be seen remotely and reduced their need for telemedicine services provided through TTP.

*And I will say that because all the doctors now have the telemed, we don't really use it that much because all of our doctors have it now because of the pandemic… They don't have it [telemedicine] after hours, but what they do have is, because it's our clinic email, the nurses can call and speak to the docs on the phone. So, then they're getting just verbal guidance.* (Director of Nursing)

### Benefits of TTP

Overall, interviewees were highly satisfied with TTP and found the program beneficial to both residents and staff. Two members of organizational-level leadership and two members of agency-level leadership called TTP a “godsend” and a valuable resource in keeping their residents out of the ED during the COVID-19 pandemic.

*We start the rollout just in time so that most of the machines were out and in place by the time the heat of the COVID hit. We didn't document it adequately because we were just doing it. We saved lives. We kept people out of the emergency rooms and provided round-the-clock telemedicine treatment options to people at just the most neediest time ever.* (Executive Vice President for Health Management Projects)

Moreover, interviewees almost universally agreed that TTP's reduction in ED visits eliminated the stress to residents generated by transportation to the ED and long wait times on arrival, and the associated costs to agencies. The TTP also enabled most residents to receive medical care in their own homes, which was particularly important in preventing possible exposure to COVID-19.

*It's excellent because… with this population, especially, it's saving such an exorbitant amount of stress on them. Some of them are very stressed to leave and get into a car, especially if they see they're going to a doctor's office or a hospital. It explodes their emotions and this is so that they're able to be seen in their own home… [W]e had many, many cases of reduced exposure just because of this, of… COVID.* (Director of Nursing)

*[W]e've been able to decrease ER use… sometimes people would wait hours and hours before they would get seen. Our folks, that's hard, particularly if they have behavioral challenges, they can sit and wait and there's staff time involved… [T]his really streamlines the medical attention process.* (Executive Director of Developmental Disability Services)

Two members of organizational-level leadership and two residential staff members emphasized StationMD's physicians' unique clinical excellence in working with PIDD.

*Well, No. 1 is they [StationMD] have I think really made extraordinary strides in the area of training their clinicians to be able to support the people with IDD, looking at the evaluation and assessment, and then the follow-up… They're extremely smart about the way that they're training their clinicians and having the clinicians interact with us.* (Executive Director)

*And when a person with a disability goes to the ER, generally what happens is the ER docs, they don't really – they don't know the individual, and if they haven't dealt with people with disabilities, they run every kind of test under the book… it's hours of the individual's time. And that's not helpful for the individual…* (Vice President, Reimbursement & Regulatory Compliance)

Two members of organizational-level leadership, five members of agency-level leadership, and one residential staff member reported that TTP minimized disruptions to residence staffing and associated costs by reducing ED visits. One to two staff members typically accompany a resident who visits the ED. To maintain state-mandated in-residence staffing ratios, agencies must pay for additional staff to fill in the gaps at the residences.

*The ER and their [residents’] behavioral needs, you may have to have two staffs… if you take them two nurses off the shift, while you've got to replace those other shifts or in the middle of the night, you're looking to bring somebody to your staffing levels that they have. So, I really do see the savings to the agency.* (Vice President)

One member of organizational-level leadership, two members of agency-level leadership, including a Nursing Director, and one Residential RN endorsed TTP as an asset to their clinical practice, reporting that it was helpful to have a second opinion from StationMD's physicians while assessing whether or not to send a resident to the ED, particularly when the resident's usual provider was unavailable.

*It's just so nice for the nurse. If she's unsure, when she gets a call, she's not quite sure how to handle it. It definitely doesn't rise to an emergency room visit, but she wants to feel good about maybe getting a second opinion, having a healthcare provider give her that confidence that she made the right decision.* (Nursing Director)

One organizational-level leader and one agency-level leader reported that notes (e.g., follow-up notes, prescriptions) from a StationMD visit were quickly uploaded and sent to the agency nurse to facilitate the nurse's follow-up with the resident. These notes were also sent to the resident's PCP to facilitate continuity of care. In some instances, StationMD's physicians directly followed up with residents after their visit.

One organizational-level leader reported instances in which StationMD's physicians directly contacted a resident's PCP when the physician observed that a chronic issue managed by the PCP (high blood sugar) was driving multiple StationMD visits or when the physician had a concern that warranted particularly close monitoring and follow-up with the PCP.

In the relatively rare cases in which a StationMD physician advised transfer to the ED, one agency-level leader noted that the physician provided a warm handoff by proactively communicating with ED clinicians in advance of the resident's arrival. This streamlined the resident's ED visit by reducing waiting time and the likelihood of unnecessary care.

The TTP also facilitated compliance with agencies' policies of ensuring medical examinations for residents within 24 h even for minor injuries or conditions (e.g., a cut, fever) by eliminating the stress and costs of in-person examination of such concerns.

### Recommended improvements to TTP

Several interviewees made suggestions to improve TTP. Two members of agency-level leadership reported a request for StationMD to offer more in-house diagnostic tools, such as remote blood sugar monitoring or EKG technology. Moreover, three members of agency-level leadership proposed changes to administrative aspects of TTP, such as making it easier to share residents' electronic health records with other telemedicine providers or providing agencies with regular reports of reasons for calling StationMD. Further, two residential staff members (DSP and Residential RN) suggested more frequent staff training on equipment use, especially given high staff turnover within residences.

### Sustainability of TTP

Interviewees, even those who did not utilize TTP frequently, universally endorsed maintaining TTP beyond the grant period. CPNYS and StationMD leadership agreed that long-term governmental funding or reimbursement for TTP is contingent on demonstrating improved health outcomes and reduced hospitalization for PIDD; less disruption to residents' routines; return on financial investment to the state; and total cost savings in Medicaid and Medicare claims. External funding is essential, because agencies' current budgets may not support sustaining TTP.

*I do think the pandemic has shown the value of telehealth, but I just hope… if we had to pay for it ourselves, I don't know that we could.* (Executive Director of Developmental Disability Services)

Further, one agency-level leader noted that agency administration may be reluctant to pay for StationMD in addition to existing on-call nursing services.

Several interviewees emphasized the importance of identifying a mechanism for agencies to bill for StationMD's services beyond the grant period. One organizational-level leader suggested a true value-based system or a waiver system (i.e., per-member, per-month fee) as potential mechanisms. Interviewees also commented that reimbursement for telemedicine needs to be at a “fair” rate; one member of organizational-level leadership stated that current Medicaid and Medicare reimbursement rates are insufficient to sustain TTP.

## Discussion

We sought to describe an innovative telemedicine program model for PIDD in group homes, and to assess utilization and staff perspectives on its broad implementation across New York State. Despite the name, it is important to note that TTP is not simply a triage model but rather provides 24/7 access to a specially trained medical provider via telemedicine, who not only determines the level of care necessary, but also provides that care in the vast majority of cases.

Over the first 2 years of its implementation, the program proved extremely feasible and acceptable, even popular, to the staff. Respondents were also highly appreciative of the special training in caring for PIDD that StationMD physicians receive which they perceived as a significant advantage over experiences in the ED.

Although originally conceived as an “after hours” service with service as well to residents remaining in the residences during the day, the advent of the COVID-19 pandemic almost immediately prompted implementers to provide the service 24/7 to all residents. Indeed, the call volume data indicate that the highest usage occurred between 10 am and 1 pm in both 2020 and 2021. The broad utilization of TTP suggests that it is filling a gap in access to care for this population, beyond providing after-hours triaging services.

A primary purpose for implementing TTP was to avert unnecessary ED visits among this vulnerable population that tends to utilize the ED more than the general population.^4,6,16–19^ Most respondents were very confident that numerous such visits had been averted. However, this preliminary assessment of TTP also indicates that many of the calls to StationMD occurred when usual care was not readily accessible for issues such as medication refill; 9% of calls in 2020 and 11% in 2021 were for this purpose.

These data suggest that TTP might be important not only for averting ED visits, but possibly UC visits as well, visits the general population makes when non-emergency care is needed quickly and usual providers are unavailable. Further, two of the most commonly used codes recorded by StationMD were “persons encountering health services in other specific circumstances” and “encounter for general adult medical examination without abnormal findings.”

These categories represented a kind of catch-all for situations that commonly arise for PIDD or in group home settings but would rarely occur in a general population, for example: staff mistakes with or questions about medication administration; situations with mandated medical clearance (e.g., a fall or altercation among residents); and staff questions about how to use adaptive devices or medical equipment.

These situations are not medical emergencies but do require professional medical attention in real time. The TTP provides such medical attention in a timely and highly efficient manner. The call data and interviews clearly demonstrated the broad use of TTP, serving as a complement in many respects to usual sources of care.

Non-emergent usage may well have been heightened by the pandemic, as clinics were closed and people were fearful leaving their homes. Interestingly though, even as the restrictions on in-person visits lessened in 2021, the call volume did not decrease, and in fact increased substantially from 2020. Respondents made clear that TTP was not a replacement for residents' usual care, but a welcome and critical complement.

The TTP program was universally well received; even staff who worked in residences that did not heavily utilize TTP thought the program was a great idea and should be continued. Consistent with prior research that identified the benefits of telemedicine delivered to PIDD,^27,28^ respondents reported that TTP significantly reduced the likelihood of encountering the stress, disruption, and costs that ED visits can present to residents and staff.

No significant obstacles or challenges were identified, other than the cost of sustaining the program beyond the funded grant period. As suggested by respondents, future improvements for the program could include offering additional diagnostic tools, providing more training for new staff, identifying mechanisms to share residents' medical records with other telemedicine providers, and providing regular reports of reasons for calling StationMD.

Research demonstrating the benefits of TTP, especially with respect to cost savings to the health care system and to the agencies themselves, will be very important in making the case to policy- and decision-makers to fund or reimburse services such as TTP.

Although this preliminary assessment provides important insights on the implementation and utilization of TTP, there are several limitations. First, residents with intellectual and developmental disabilities and/or their families were not interviewed because that was outside the scope of work and as such, their perspectives were not included in this assessment.

There were plans to informally speak with residents during in-person site visits to select group residences, but these site visits could not be completed due to COVID-19 pandemic restrictions. As a result, this preliminary assessment includes only leadership and staff perspectives on the implementation of TTP and may not fully capture the experiences of residents using the telemedicine service. It is also possible that the purposive, voluntary sample underrepresents staff who did not view the program favorably.

## Conclusions

The results from this preliminary assessment of utilization and staff perspectives suggest that the TTP model is an acceptable and feasible way to provide equitable access to telemedicine for PIDD that complements in-person health care, an under-resourced area that demands more attention.^27^ This model addresses the barriers to and disparities in access to health care that affect PIDD, including long ED wait times,^4,5^ inaccessible medical facilities, discriminatory or insufficiently culturally competent providers,^3,5–9^ and unavailability of individualized accommodations.^6,10^

The COVID-19 pandemic clearly facilitated the implementation of TTP and likely increased the initial use of the program. Future research is needed to determine how usage of the program plays out post-pandemic. Since this preliminary assessment focused on the utilization of TTP early in its implementation phase, additional interviews could assess the program in its more mature phase.

Future research should also explore residents' experiences with using TTP and interacting with StationMD physicians on a telemedicine consultation to determine what works well and what can be improved. Finally, research demonstrating the effectiveness of TPP in averting ED and UC visits is urgently needed.

## Supplementary Material

Supplemental data

## Abbreviations Used

CPNYS: Cerebral Palsy Associations of New York State
DSPs: direct support professionals
ED: emergency department
ICD: International Classification of Diseases
PCPs: primary care physicians
PIDD: people with intellectual and developmental disabilities
TTP: Telemedicine Triage Project
UC: urgent care

## Notes

^†^
Urgent care is defined as the provision of immediate medical service offering outpatient care for the treatment of acute and chronic illness and injury. https://aaucm.org/what-is-urgent-care-medicine/

^‡^
Despite the misnomer, we use the official name of the program to be consistent with its grant funding and for all those involved who know it by this name.
