# Supplementary material for: Utilization and Staff Perspectives on an On-Demand Telemedicine Model for People with Intellectual and Developmental Disabilities Who Reside in Certified Group Residences
Source: Telemed Rep. 2023 Jul 31;4(1):204–14. doi: 10.1089/tmr.2023.0024 (PMC10389255; doi:10.1089/tmr.2023.0024)
Supplement: Supplemental data [file Suppl_FileS1.docx]

**Interview Protocol - StationMD**

**Respondent Background**

What is your role at the Station MD? How long have you been in your position? How long have you been with organization?

What is your current role with respect to the TTP initiative?

**TTP Development/Planning**

Were you involved in its development and design of TTP? *If yes,* What was your role in that process?

*If no, skip to* ***Early Implementation****.*

Do you know what led to the decision to plan and implement TTP? What were the motivating factors?

Who was involved in the early decision-making process? How did you decide which staff needed to be involved in designing and/or planning TTP? Were they the right people? Were residential staff involved in the planning? Was Station MD involved?

In retrospect, do you wish you had involved anyone else?

What were the initial goals for the TTP pilot? Have those goals changed?

**Early Implementation**

How was Station MD selected as the triage vendor? Were other organizations considered? Why was Station MD the one selected? When did Station MD become involved?

Tell me how TTP was supposed to operate. Did you develop a centralized protocol or did residences work out their own processes?

What were the challenges to launching the TTP pilot and how were those challenges addressed? What factors were critical to successful initial launch?

What have you learned from the early stages of implementation? What changes were made to operations, if any?

**Current Operations**

How does TTP work now?

Is TTP operating in all CP residences in NYS? If not, why not?

What do you know about the current volume of TTP calls?

Do you have the sense that some residences are using it more than others? If yes, what are the factors driving the differences in uptake?

How does Station MD’s work with TTP differ from your work with other clients?

How did COVID-19 affect TTP? What changes, if any, were made to the protocols/workflows?

Did COVID-19 change the volume of calls?

PROMPT: It was mentioned in a meeting that COVID-19 was perhaps causing a shift from triage only to telehealth visits. Could you elaborate on that?

**Results**

What has the impact been on residences? Has there been any effects, positive or negative, on staff? Has it affected job satisfaction?

Have you seen evidence of program effects so far? What are the effects on residents? Families?

What type of empirical evidence would you like to see to demonstrate the success of TTP?

Are there changes or improvements you would like to see to the TTP initiative? Do you think the TTP is sufficiently comprehensive? If not, what would make it so?

**Sustainability**

Do you think TTP should continue? Why or why not?

What might derail the program in the future?

What are the critical factors to ensure that it will continue?

Is there anything else you would like to share about your experience with this initiative?

**Interview Protocol – CPNYS**

**Respondent Background**

What is your role at the CP-NYS? How long have you been in your position? How long have you been with organization?

What is your current role with respect to the TTP initiative?

**TTP Development/Planning**

Were you involved in its development and design of TTP? *If yes,* What was your role in that process?

*If no, skip to* ***Current Operations****.*

What kind of triage process, if any, was happening at the residential facilities prior to this initiative?

Was there any type of telehealth/telemedicine occurring at the residences?

What led to the decision to plan and implement TTP? What were the motivating factors?

Who was involved in the early decision-making process? How did you decide which staff needed to be involved in designing and/or planning TTP? Were they the right people? Were residential staff involved in the planning? Was Station MD involved?

In retrospect, do you wish you had involved anyone else?

What were the initial goals for the TTP pilot? Have those goals changed?

How was Station MD selected as the triage vendor? Were other organizations considered? Why was Station MD the one selected? When did Station MD become involved?

How were residential staff informed of the new program?

Tell me how TTP was supposed to operate. Did you develop a centralized protocol or did residences work out their own processes?

What were the challenges to launching the TTP pilot and how were those challenges addressed? What factors were critical to successful initial launch?

What have you learned from the early stages of implementation? What changes were made to operations, if any?

**Current Operations**

How does TTP work now?

Is TTP operating in all CP residences in NYS? If not, why not?

What do you know about the current volume of TTP calls?

Do you have the sense that some residences are using it more than others? If yes, what are the factors driving the differences in uptake?

How did COVID-19 affect TTP? What changes, if any, were made to the protocols/workflows?

Did COVID-19 change the volume of calls?

PROMPT: It was mentioned in a meeting that COVID-19 was perhaps causing a shift from triage only to telehealth visits. Could you elaborate on that?

**Results**

What has the impact been on residences? Has there been any effects, positive or negative, on staff? Has it affected job satisfaction?

Have you seen evidence of program effects so far? What are the effects on residents? Families?

What type of empirical evidence would you like to see to demonstrate the success of TTP?

Are there changes or improvements you would like to see to the TTP initiative? Do you think the TTP is sufficiently comprehensive? If not, what would make it so?

**Sustainability**

Do you think TTP should continue? Why or why not?

What might derail the program in the future?

What are the critical factors to ensure that it will continue?

Is there anything else you would like to share about your experience with this initiative?

**Interview Protocol – Agency-level Leadership and Residential Staff**

**Respondent Background**

What is your role at the [*residence name*]? How long have you been in your position? How long have you been with the organization?

What is the nature of your interaction with residents? With other staff?

**Prior to TTP/Program Development**

What kind of triage process, if any, was happening at this residential facility prior to TTP? How were medical situations handled? What were your responsibilities with respect to facilitating medical services and care for residents?

Was there any type of telehealth/telemedicine occurring at the residences? *If yes*, for what types of services? What, if any, role did you play?

Were you involved in its development and design of TTP? *If yes,* What was your role in that process?

What were the initial goals for the TTP pilot? Have those goals changed?

*If not involved in planning*, how did you first learn about the new tele triage program? What did you understand to be the purpose or goals of TTP?

**Current Operations**

How does TTP work now?

What type of situation warrants a call to TTP?

Who makes the decision to call TTP?

Do residents know about TTP?

Do you need consent from residents/families?

About how many calls a week are made from this residence to the TTP? Has that changed over time?

Did you receive any training on the TTP? Did other staff at your facility?

How often have you placed a call to TTP? Tell me about some of your experiences using TTP.

How quickly do you typically get through?

How responsive are the doctors on the line?

How competent do the doctors seem to you?

Have you been satisfied with the triage decisions?

What challenges did you face in implementing TTP here?

How did COVID-19 affect TTP?

Did COVID-19 change the volume of calls to TTP? *If yes*, why/how?

How has COVID-19 affected operations at this residence in general? *If yes*, why/how?

**Results**

What do you think about TTP? How has it affected operations here? Would you say it has been successful?

How has it affected your job?

What are the effects on residents? Families? Other staff?

What type of empirical evidence, if any, would you like to see to demonstrate the success of TTP?

Are there changes or improvements you would like to see to the TTP initiative? Do you think the TTP is sufficiently comprehensive? If not, what would make it so?

**Sustainability**

Do you think TTP should continue? Why or why not?

What might derail the program in the future?

What are the critical factors to ensure that it will continue?

Is there anything else you would like to share about your experience with this initiative?
